# Error rates for unvalidated medical age assessment procedures

**DOI:** 10.1007/s00414-018-1916-3

**Published:** 2018-09-15

**Authors:** Petter Mostad, Fredrik Tamsen

**Affiliations:** 1 Mathematical Sciences, Chalmers University of Technology, Gothenborg, Sweden; 2 Mathematical Sciences, Gothenburg University, Gothenburg, Sweden; 3 Forensic Medicine, Department of Surgical Sciences, Uppsala University, Uppsala, Sweden

**Keywords:** Medical age assessment, Third molar, Femur, Knee, Bayesian

## Abstract

During 2014–2015, Sweden received asylum applications from more than 240,000 people, of which more than 40,000 were termed unaccompanied minors. In a large number of cases, claims by asylum seekers of being below 18 years were not trusted by Swedish authorities. To handle the situation, the Swedish national board of forensic medicine (Rättsmedicinalverket, RMV) was assigned by the government to create a centralized system for medical age assessments. RMV introduced a procedure including two biological age indicators; x-ray of the third molars and magnetic resonance imaging of the distal femoral epiphysis. In 2017, a total of 9617 males and 337 females were subjected to this procedure. No validation study for the procedure was however published, and the observed number of cases with different maturity combinations in teeth and femur were unexpected given the claims originally made by RMV. We present a general stochastic model enabling us to study which combinations of age indicator model parameters and age population profiles are consistent with the observed 2017 data for males. We find that, contrary to some RMV claims, maturity of the femur, as observed by RMV, appears on average well before maturity of teeth. According to our estimates, approximately 15% of the tested males were children. These children had an approximate 33% risk of being classified as adults. The corresponding risk for an adult to be misclassified as a child was approximately 7%. We determine uncertainties and ranges of estimates under reasonable perturbations of the prior.

## Introduction

In medical age assessments, certain biological processes that develop during childhood in a predictable sequence are used to assess a person’s chronological age. Examples of such processes are the development of teeth, bones, and sexual maturity. We will use in this paper the term ‘age indicator” to mean any observed biological feature that develops through a series of clearly defined states over an age period relevant for age assessment.

Medical age assessments have been used for a long time in many countries, but are always associated with debate. This debate can be divided into two main lines. One concerns the very use of medical age assessment. Critics argue that since biological processes always show such a wide variation in populations, biological states can never be used to make sufficiently certain assessments of chronological age. Others might argue that it is the biological and not the chronological age that is the most relevant for the needs of an individual.

In most countries, the age is important for the rule of law. Many countries view the age of 18 as being the border between childhood and adulthood. Children have other needs and rights than do adults, and punishments for crimes might differ whether the perpetrator is below or above 18. In the case of asylum seekers, children are to be treated differently according to international conventions. One might therefore argue that if a person’s age is unknown, a medical age assessment may be necessary in order to protect the privileges of children.

The other line of debate concerns which methods are appropriate to use. A compilation of methods used in the European Union shows that most countries use two or more age indicators [3]. There are variations between countries, but the two most commonly used methods are dental x-ray and x-ray imaging of the hand/wrist. Another commonly used indicator is x-ray imaging of the collar bone. These three age indicators are all included in the recommendations by The Study Group on Forensic Age Diagnostics (Arbeitsgemeinschaft für Forensische Alters- diagnostik; AGFAD), an international assembly of experts that has worked on this issue for the last 18 years [16, 17].

During the last few years, many asylum seekers in Sweden have claimed to be under 18 but have not been able to convince authorities about their claim. To be able to treat children as children, and to not give child privileges to adults, the government of Sweden has decided to offer the possibility to make a medical age assessment in these cases. When a wave of asylum seekers arrived in 2014–2015, there was no generally accepted system for medical ages assessments in Sweden, and Rättsmedicinalverket (RMV) was assigned by the government to create one.

The method^1^ chosen by RMV uses two age indicators: magnetic resonance imaging of the distal femur (MRI knee) and x-ray imaging of the third molars in the mandible (x-ray teeth) [14]. The MRI knee and x-ray teeth are independently evaluated by two radiologists and two dentists, respectively. For the knee to be assessed as mature, both radiologists must agree on this assessment. If they disagree, the knee is assessed as immature. The same procedure is used for teeth. These assessments are then combined in such a way that if either the knee or the teeth are mature, a male individual is assessed as being 18 years or older^2^. During 2017, a total of 9617 males and 337 females were subjected to this age assessment procedure. The results for males are given in Table 1. In 2018, RMV changed their assessments for females, since a new study showed that the majority of females aged 16 and 17 years had mature knees (see Ottow et al. [12], Tamsen [19]). Females now need mature knees and teeth to be assessed as being 18 years or older. In this paper, we will only study the RMV data for males.

**Table 1:** Results for the 9280 males submitted to the RMV procedure during 2017

	Knees mature	Knees immature	No data knees	Sum
Teeth mature	4176	348	187	4711
Teeth immature	1735	1087	83	2905
No data teeth	1364	237	63	1664
Sum	7275	1672	333	9280

The maturity of the teeth is assessed according to the stages of Demirjian, in which a tooth can be in one of eight stages A–H (see Demirjian et al. [2]). H is the final stage and the teeth are termed “mature” if at least one of the mandibular third molars are assessed as being in this stage. The knee is assessed as “mature” if it has reached stage 4 or 5 according to the classification by Schmeling [8]. The different stages of immature teeth and knees are not used in the age assessments made by RMV. For example, if the knee is mature, it doesn’t matter if the most developed examined tooth is in stage G (one stage from mature) or stage F (two stages from mature), the age assessment is still the same.

To date, there exist six original articles and one letter to the editor on age assessment with MRI knee: Dedouit et al. [1], Krämer et al. [8], Saint-Martin et al. [15], Ekizoglu et al. [4], Fan et al. [5], Ottow et al. [12], Vieth et al. [23]. In addition, there is a non-peer-reviewed report published by The National Board of Health and Welfare (Socialstyrelsen) in Sweden [18]. Since there are differences in MRI techniques and grading systems for maturity assessment, studies differ as to how relevant they are in relation to the RMV procedure. Three of the original studies use MRI techniques and grading systems that are more or less comparable to the method used by RMV [5, 8, 12]. However, the relatively small number of participants in relevant ages and shifting results make it hard to regard this method as validated. More and larger studies are needed.

Another aspect of validity is the application of the methods. Validation of assessments is an obvious practice in the field of medicine. Normally, an apprentice makes assessments under the supervision of an experienced assessor. When the rate of correct assessments is sufficiently high, the apprentice is allowed to make them on his or her own. At least for the maturity assessments of MRI knee, RMV has not presented any external validation prior to the large amount of assessments they now have performed. We also believe a validation should include a study where one applies to volunteers with known ages the exact same assessment procedure as the one applied to subjects, including both knee and teeth assessments and using the same assessors.

In 137 cases where RMV assessed the knee as mature, an external second opinion has been performed by German scientists^3^. These scientists are the ones who have developed and continued to study an MRI knee method close to the one RMV uses. In 75 of these 137 cases (55%), the German scientists came to the opposite conclusion that the knee was not mature. The cases that have undergone second opinions are the result of private initiatives and they are thus not randomly selected. Therefore, one cannot generalize these results to all people who have had their knees assessed as mature. However, one cannot exclude general discrepancies and since RMV uses these German studies as the most important foundation for their method, the results are alarming and require a thorough analysis of the validity of the Swedish assessments. When faced with this criticism, RMV performed an analysis of reliability, but no analysis of validity has yet been reported.

We are thus facing a situation where there is substantial uncertainty about the true relationship between chronological age and the age indicators used by RMV. There is of course, also a large uncertainty about the true age distribution of the population on which the procedure has been performed. The only firm evidence is the information presented in Table 1. Note that according to the table, about five times as many males are classified with a mature knee and immature teeth compared to vice versa. This seems at odds with earlier statements from RMV that knees generally mature later than teeth. In this paper, we show how simulation within a Bayesian framework may be used to obtain information about the possible combinations of population age profiles and age indicator models that may explain this data. We also show how one may obtain some information about likely classification error rates in such a situation.

A simple statistical approach to medical age assessment is the following: an age indicator that can take on discrete values $I_{1},\dots , I_{n}$ is measured on a study population with known chronological ages. The study population is subdivided according to the age indicator, and the chronological ages within each subgroup are modeled with some statistical model, possibly just a normal distribution. Then, this statistical model is used to assess the chronological age of persons whose observed age indicator corresponds to the group.

The main drawback of this simple and common approach is that it assumes that, a priori, the distribution of the ages of the assessed persons corresponds to the distribution of ages in the study population. This is clearly not the case in most applications of age assessments, as the assessment is generally triggered by circumstances related to age. For example, an immigration authority may decide to require medical age assessment of all asylum seekers, of asylum seekers they believe might be re-classified by the assessment, or of asylum seekers whose age they are fully convinced are above the relevant age limit of 18 years. Clearly different decisions will lead to different rates of erroneous classification, something that cannot be captured by the simple statistical procedure above.

In this paper, we instead use the following procedure: For each age indicator, we use studies where the indicator has been observed in study populations with known chronological ages to establish a statistical model predicting the value of the age indicator as a function of chronological age. When assessing the age of a person with an observed age indicator, we combine an a priori distribution for the persons age with the likelihood provided from the age indicator and the statistical model to obtain the a posteriori distribution for the age of the person. This Bayesian approach is discussed for example in Taroni et al. [20] (relating to forensics in general) and for example in Thevissen et al. [21] (relating to age assessments).

For each of the two age indicators appearing in this paper, we thus need to establish a statistical model predicting the value of the age indicator from chronological age. General models are discussed in “Stochastic model”. How to obtain model parameters from published studies is discussed in “Age indicator model parameter values”. In this paper, we assume that, given chronological age, the probability for observing various values of one indicator is independent of the value observed for another indicator. Such an assumption is an approximation of reality, and one needs to ask how large the approximation is, if it can be avoided, and in what way it may influence results. Conditional correlation of age indicators has not been much studied, but Gelbrich et al. [6] found no significant such correlation between wrist and third molar maturation. No study has investigated the conditional correlation between knee and third molar maturation, indicating again the need for a proper validation of the RMV procedure. As no data to build models exists, we are forced in this paper to assume conditional independence between knee and third molar maturation. Possible consequences of this assumption are discussed in “Discussion”.

In order to investigate which combinations of age indicator models and population age profiles can explain the data of Table 1, we need to establish an a priori distribution for the age of the person that is assessed. In case work, such a distribution will be based on the circumstances of that person and may vary from case to case. In this paper, we consider data derived from age assessment of 9280 males, and we use a common a priori distribution for these, based simply on the fact that they have in a sense been required^4^ to submit themselves to the medical age assessment procedure arranged by RMV in Sweden.

Preliminary computations presented in our supplementary material [11] show that using fixed age indicator models with parameters directly derived from relevant publications together with a fixed population age profile generally yields models that cannot explain the data of Table 1. Thus, we instead build a prior model for the age indicators (“Age indicator model parameter values”) and for population age profile (“Specification of prior for the population profile”) and study predictions from the posterior model given this data (“Results”). In particular, we study the general properties of the RMV procedure viewed as a classification method and draw some conclusions about error rates. We also study how robust these conclusions are under reasonable changes in the prior (“Robustness”).

## Methods

In “Stochastic model”, we present the stochastic model enabling us to do the computations specified above. In “Age indicator model parameter values”, we present the models we use for teeth and knee age indicators while “Specification of prior for the population profile” contains a discussion on how we model the age distribution. Finally, “Convergence and accuracy” contains some technical information surrounding simulation with our model.

### Stochastic model

We assume *K* different age indicators are observed. We assume age indicator *k* ($k = 1,\dots ,K$) can take on *n*_*k*_ different discrete values, denoted $I_{k1},I_{k2},\dots ,I_{kn_{k}}$. For each age indicator *k*, we assume there is a model with parameters *𝜃*_*k*_ relating the chronological age *x* of a person to the probabilities *p*_*kj*_(*x*∣*𝜃*_*k*_) of observing indicator *I*_*kj*_, so that we assume

1 $$ \sum_{j = 1}^{k_{i}}p_{kj}(x\mid\theta_{k})= 1 $$

for all *x*.

As an example, assume age indicator *k* has two different values, *I*_*k*1_ representing “immature” and *I*_*k*2_ representing “mature”. In some cases, a reasonable parametric model may be

2 $$ p_{k2}(x\mid\theta_{k})= {\Phi}\left( \frac{x - \theta_{k1}}{\theta_{k2}}\right)  $$

where *𝜃*_*k*_ = (*𝜃*_*k*1_,*𝜃*_*k*2_) and Φ is the inverse Probit function (i.e., the cumulative distribution function for the standard normal distribution). Note that *𝜃*_*k*1_ then represents the age at which 50% of all persons have attained the age indicator *I*_2_, while *𝜃*_*k*2_ represents the variation of the age of attainment around *𝜃*_*k*1_. We will consider models where age indicator *k* can have a third possible value, *I*_*k*3_, representing “not assessible”. In fact, a model with a constant probability for such missing data does not fit the data considered in this paper. Thus, we use instead a linear dependency of lack of data on age:

3 $$\begin{array}{@{}rcl@{}} p_{k3}(x\mid\theta_{k}) &=& \theta_{k3} + \theta_{k4}(x-20) \end{array} $$

4 $$\begin{array}{@{}rcl@{}} p_{k2}(x\mid\theta_{k}) &=&\left( 1-p_{k3}(x\mid\theta_{k})\right){\Phi}\left( \frac{x-\theta_{k1}}{\theta_{k2}}\right) \end{array} $$

where now *𝜃*_*k*_ = (*𝜃*_*k*1_,*𝜃*_*k*2_,*𝜃*_*k*3_,*𝜃*_*k*4_).

For each age indicator *k*, we use a probability density on the space of possible parameters *𝜃*_*k*_ to model the uncertainty in the model. Specifically, consider the model of Eqs. 3 and 4. As it is reasonable to think that, given age, lack of data is independent of maturity of the indicator, we may write

5 $$ \pi(\theta_{k}) = \pi(\theta_{k1},\theta_{k2})\pi(\theta_{k3},\theta_{k4}).  $$

In our setting, the parameters *𝜃*_*k*3_ and *𝜃*_*k*4_ concerning lack of data will be well informed by the data we are considering, so we will use flat priors *π*(*𝜃*_*k*3_,*𝜃*_*k*4_) ∝ 1 for these. The priors *π*(*𝜃*_*k*1_,*𝜃*_*k*2_) will be based on information obtained from various published studies and will be further discussed in “Age indicator model parameter values”. We now define a joint prior

6 $$ \pi(\theta) = \pi(\theta_{1})\pi(\theta_{2})\dots\pi(\theta_{K}) $$

where $\theta = (\theta _{1},\dots ,\theta _{K})$.

As mentioned above, we assume in this paper that, given chronological age *x*, the probability for observing various values of one indicator is independent of the value observed for another indicator. Thus, assuming a person has age *x*, that a vector $v=(z_{1},\dots ,z_{K})$ of the *K* different age indicators is observed for this person, and given a value for *𝜃*, the probability of observing *v* can be written as

7 $$ p(v\mid x,\theta) = \prod_{k = 1}^{K} p_{kz_{k}}(x\mid\theta_{k}). $$

Aside from the parameters of the age indicator observation models used, the major uncertainty in our situation lies in the distribution of chronological ages in the population on which the observation procedure is applied. In this paper, we will use a discretization, using the vector $\{x_{1},\dots ,x_{T}\}$ to represent *T* possible age values. A population profile is then represented by a vector $\psi =(\psi _{1},\dots ,\psi _{T})$, with *ψ*_*i*_ indicating the probability for age *x*_*i*_, so that $\sum _{i}\psi _{i}= 1$. We will use a Dirichlet prior on *ψ*, with

8 $$ (\psi_{1},\dots,\psi_{T})\sim\operatorname{Dirichlet}(\alpha/T, \dots,\alpha/T). $$

for some parameter *α*. Under this prior, the expected value of each *ψ*_*i*_ is 1/*T*. Starting with some distribution with cumulative density function *F* which can be considered reasonable, we choose the *x*_*i*_ so that *F*(*x*_*i*_) = *i*/*T*. Thus the uneven spread of the *x*_*i*_ will reflect the population profile specified by *F*. The uncertainty around this target distribution is governed by the parameter *α*: We get that

9 $$ \psi_{1}+\dots+\psi_{i} \sim\operatorname{Beta}\left( \frac{i}{T}\alpha, \frac{T-i}{T}\alpha\right) $$

so that when $\alpha \to \infty $, we get increasingly little variation around the target distribution, while *α* → 0 gives increasing flexibility.

To make computations, we include in the model a variable with information about the actual ages of the persons subjected to the age assessment procedure. Let $v_{1},v_{2},\dots ,v_{V}$ be the possible values that an age indicator vector *v* can take on, so that *V* = *n*_1_*n*_2_⋯*n*_*K*_. Now let *τ*(*v*_*i*_,*x*_*j*_) represent the count of persons of age *x*_*j*_ having observational vector *v*_*i*_, and let

10 $$ \tau = \left\{\tau(v_{i},x_{j})\right\}_{i = 1,\dots,V; j = 1,\dots,T} $$

so that *τ* is the collection of all these counts. Fixing *𝜃* and *ψ*, *τ* has a multinomial distribution,

11 $$\begin{array}{@{}rcl@{}} \tau\mid\theta,\psi&\sim&\operatorname{Multinomial}\\&&\times\left( N, \left\{r(v_{i},x_{j}\mid\theta,\psi)\right\}_{ i = 1,\dots,V; j = 1,\dots,T}\right) \end{array} $$

where *N* is the total number of persons observed and

12 $$ r(v_{i},x_{j}\mid\theta,\psi) = \psi_{i} p(v_{i}\mid x_{j},\theta) $$

is the probability that a person has age *x*_*j*_ and observational vector *v*_*i*_.

The actual observations are contained in the vector $y=(y_{1},\dots ,y_{V})$ where, for $i = 1,\dots ,V$,

13 $$ y_{i} = \sum\limits_{j = 1}^{T}\tau(v_{i}, x_{j}). $$

We have now formulated a full stochastic model for our variables:

14 $$ \pi(y,\tau,\theta,\psi) = \pi(y\mid\tau)\pi(\tau\mid\theta,\psi)\pi(\theta)\pi(\psi). $$

Our strategy is to simulate from this joint distribution conditional on the observed data *y* using the Metropolis-Hastings algorithm. There are three different updating steps, where each of the variables *τ*, *𝜃*, and *ψ* are updated while the other variables are kept fixed.

For *τ*, we get

15 $$ \pi(\tau\mid y, \theta,\psi) \propto \pi(y\mid\tau)\pi(\tau\mid\theta,\psi) $$

and as *π*(*y*∣*τ*) simply restricts the sums of counts in *τ*, we get for $i = 1,\dots ,V$ that

16 $$\begin{array}{@{}rcl@{}} &&\left( \tau(v_{i},x_{1}),\dots,\tau(v_{i},x_{T})\right)\sim\operatorname{Multinomial}\\&&\times\left( y_{i}, \left\{\frac{r(v_{i},x_{j}\mid\theta,\psi)}{\sum_{k = 1}^{T}r(v_{i},x_{k}\mid\theta,\psi)}\right\}_{j = 1,\dots,T}\right). \end{array} $$

For *𝜃*, we get

17 $$\begin{array}{@{}rcl@{}} \pi(\theta\mid y,\tau,\psi) \!&\propto &\! \pi(\tau\mid\theta,\psi)\pi(\theta) \\ &\propto &\!\left[\!\prod\limits_{i = 1}^{V}\prod\limits_{j = 1}^{T}r(v_{i},x_{j}\mid\theta,\psi)^{\tau(v_{i},x_{j})}\!\right] \prod\limits_{k = 1}^{K}\pi(\theta_{k})\\ &\propto&\!\left[\prod\limits_{i = 1}^{V}\prod\limits_{j = 1}^{T}p(v_{i}\mid x_{j},\theta)^{\tau(v_{i},x_{j})}\right] \prod\limits_{k = 1}^{K}\pi(\theta_{k})\\ \end{array} $$

which splits as a product over the different age indicators:

18 $$ \pi(\theta_{k}\mid y,\tau,\psi) \propto\pi(\theta_{k})\prod\limits_{z_{k}= 1}^{n_{k}}\prod\limits_{j = 1}^{T}p_{kz_{k}}(x_{j}\mid \theta_{k})^{\tau^{\prime}(z_{k}, x_{j})} $$

where

19 $$ \tau^{\prime}(z_{k},x_{j}) = \sum\limits_{z_{1}= 1}^{n_{1}}\dots\sum\limits_{z_{k-1}= 1}^{n_{k-1}}\sum\limits_{z_{k + 1}= 1}^{n_{k + 1}}\dots\sum\limits_{z_{K}= 1}^{n_{K}} \tau((z_{1},\dots,z_{K}),x_{j}). $$

In other words, the posterior probability for a parameter vector *𝜃*_*k*_ is proportional to its prior probability times the product of the probabilities of observing each of the *n*_*i*_ indicator values at each of the possible ages to the power of the count of the persons having this age and indicator value.

Using a random walk proposal function in the Metropolis-Hastings procedure, we can calculate the acceptance probability at each stage. (See “Convergence and accuracy” for details.)

For *ψ*, we get

20 $$\begin{array}{@{}rcl@{}} \pi(\psi\mid y, \tau,\theta) &\propto& \pi(\tau\mid\theta,\psi)\pi(\psi) \\ &\propto& \pi(\psi)\prod\limits_{j = 1}^{T}q(x_{j}\mid\psi)^{\tau^{\prime\prime}(x_{j})} \\&=& \pi(\psi)\prod\limits_{j = 1}^{T}\psi_{j}^{\tau^{\prime\prime}(x_{j})} \end{array} $$

where

21 $$ \tau^{\prime\prime}(x_{j}) = \sum\limits_{v_{i}= 1}^{V}\tau(v_{i},x_{j}). $$

Using the Dirichlet prior *π*(*ψ*) mentioned above, we may simulate *ψ* from

22 $$ \psi\mid\tau\sim\operatorname{Dirichlet}\left( \tau^{\prime\prime}(x_{1})+\alpha/T,\dots,\tau^{\prime\prime}(x_{T})+\alpha/T\right). $$

### Age indicator model parameter values

We now turn to obtaining estimates $\hat {\theta }_{k1}$ and $\hat {\theta }_{k2}$ for the parameters of Eq. 2 from published studies on age indicators. Recall that *𝜃*_11_ represents the age at which 50% have attained mature molars among those males whose molar stage can be measured, while *𝜃*_12_ represents the variability in this age of attainment. For example, according to the model, the proportions with mature molars will be approximately 16% and 84% at ages *𝜃*_11_ − *𝜃*_12_ and *𝜃*_11_ + *𝜃*_12_, respectively. The parameters *𝜃*_21_ and *𝜃*_22_ represent corresponding values for knee maturity.

Given the raw data from an age indicator study, i.e., a list of pairs of observed chronological ages and age indicators, one may use maximum likelihood to fit a model like that of Eq. 2 and thus obtain an estimate for the model parameters. However, age indicator studies tend not to publish their raw data and a more indirect approach is necessary. We have chosen to in each case construct a plausible raw data set based on the information in the paper, and then estimate parameters based on this. As ways of obtaining such raw data is not the main focus of this paper, we have chosen fairly ad hoc procedures.

The DARLInG reference data sets, e.g., UK-caucasian [22], may be as close as one can get to obtaining publicly available raw data about tooth age indicators. We have taken from this database information about the ages of the 591 males with lower left third molars in stages D through H. For each maturity stage, we have assigned ages according to a normal distribution, applying afterwards a piecewise linear transformation to map the quantiles of this distribution to the quantiles listed for the maturity stage. Fitting the model of Eq. 2 to this data using maximum likelihood, we obtain the estimates listed in Table 2.

**Table 2:** Parameters for age indicator models: estimates and priors. *𝜃*_11_ represents the age at which 50% of males have mature third molars

	DARL	Lucas	Mincer	Haglund	Prior		Soc.s.	Ottow	Adj. Ott.	Prior
*𝜃*_11_	19.5	18.6	19.9	20.9	19.5	*𝜃*_21_	18.5	18.5	17.7	18.5
*𝜃*_12_	1.6	0.8	2.2	2.5	1.6	*𝜃*_22_	1.3	1.5	1.4	1.4

At the ages *𝜃*_11_ − *𝜃*_12_ and *𝜃*_11_ + *𝜃*_12_, 16% respectively 84% have mature third molars. The first four columns display estimates of these values based on four different studies, while the fifth column indicates the values we have chosen in our prior as the most likely for the RMV procedure. The right-hand part of the table displays similar values for knee maturity

In Lucas et al. [9], examinations from a total of 1000 males are reported, subdivided according to age into 20 groups of 50 males each. Each group consists of persons with ages within a specified half-year interval; we approximated the ages as uniformly spread within these intervals. Table 2 in Lucas et al. [9] reports the number of males within each group that have mature teeth^5^. We have randomly selected the corresponding number of males in each age group and assigned them an observation of mature teeth, thus creating data that should correspond fairly well to the RMV procedure. Applying maximum likelihood estimation, we obtain the estimates shown in Table 2.

In the classic paper Mincer et al. [10], the male study population consists of 271 individuals. The number of males observed with each of the age indicators D, E, F, G, and H for the mandible is not reported, but we interpolate these values from Table 1 in the paper, obtaining 37, 43, 45, 55, and 91, respectively. The quantiles of the ages of the persons in each of these five groups are reported in Table 3 of the paper. Using the same technique as for the DARLInG data set, we reconstruct plausible raw data based on this information and obtain estimates listed in Table 2.

**Table 3:** Posterior results using the main prior. The table shows the expected number of people within each group. The parentheses show 95% credibility intervals

	Classified as adults	Classified as children	Not classified	Sum
Adults	7260 (5908 – 7794)	581 (116 –1305)	59 (49 –63)	7900 (6102–8570)
Children	550 (16 – 1902)	826 (102 –1291)	4 (0 – 14)	1380 (133 –3379)
Sum	7810	1407	63	9280

Finally, a recent study [7] pools information from a large number of studies where the third molar has been used as an age indicator. Table 2 in that paper contains age information from a total of 11,832 persons^6^. In a similar way as for the the data in Lucas et al. [9] we have assigned exact ages to these 11,832 persons uniformly within the age intervals and randomly selected the indicated proportion of these as having mature teeth. We then used maximum likelihood to obtain estimates *𝜃*_11_ = 20.9 and *𝜃*_12_ = 2.5.

Note that there are substantial differences between estimates based on different studies. Substantial differences have also been previously noted (see, e.g., Rolseth et al. [13] page 111). As an example, Table 2 of Haglund and Mörnstad [7] indicates that 9.4% of 381 25-year olds with a determinable third molar stage have a stage below H. This contrasts with Lucas et al. [9] where 100% of 1200 persons between 20 and 25 have stage H. A possible cause is that studies vary in their details, differing from the RMV procedure to various degrees. There may also be systematic biases between studies caused by the use of different medical personnel trained in different ways.

Conclusions for the parameters of the RMV procedure are thus uncertain, and to model this, we use a prior with a substantial amount of uncertainty. Specifically, our prior density for the parameters *𝜃*_11_ and *𝜃*_12_ of the RMV procedure is

23 $$\begin{array}{@{}rcl@{}} \pi_{T_{\text{MAIN}}}(\theta_{11},\theta_{12}) &\propto& \operatorname{Normal}(\theta_{11}; 19.5, 0.8) \cdot\\&&\operatorname{Normal}(\theta_{12}; 1.6, 0.4)\cdot I(\theta_{12}>0).\\ \end{array} $$

**Fig. 1:**
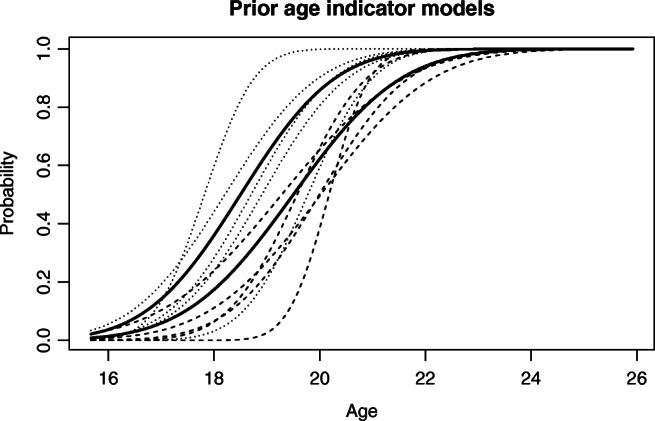
The prior probability for observing mature teeth (right bold line) or knees (left bold line) as a function of age, assuming the observation is not missing. The two bold lines represent the most likely models. The 5 dashed lines represent other possible prior models for teeth maturity, while the 5 dotted lines represent possible prior models for knee maturity

In other words, the prior density is proportional to the product of two normal densities, truncated so that *𝜃*_12_ is positive. The numbers 0.8 and 0.4 appearing in the formula above indicate the variability of the prior. A way to understand the prior is that we are approximately 95% sure *𝜃*_11_ is in the interval [19.5 − 2 ⋅ 0.8,19.5 + 2 ⋅ 0.8] and, independently, approximately 95% sure *𝜃*_12_ is in the interval [1.6 − 2 ⋅ 0.4,1.6 + 2 ⋅ 0.4]. The prior is illustrated in Fig. 1. We have chosen to center the prior on the estimates from the DARLInG dataset: This data refers exactly to the lower left third molars for males used in the RMV procedure, and the parameter estimates are in between estimates based on other data. In “Robustness”, we have investigated the robustness of our main conclusions under reasonable changes to this prior: Calling the prior above *T*_MAIN_, we have also looked at results using priors *T*_HIGH_ and *T*_LOW_ which differ from *T*_MAIN_ in that the the expected value of *𝜃*_11_, 19.5, is replaced by 20.5 or 18.5, respectively (see “Robustness”). In our supplementary material [11], we confirm that our main conclusions hold even when using a prior centered on the Haglund estimates.

Turning to knees and the parameters *𝜃*_21_ and *𝜃*_22_, one source of information is Socialstyrelsen [18], which reports on two methods for assessing age from knee MRI images. The study has, to our knowledge, not yet been peer reviewed, but has been published as a report. One of the methods investigated is designed to correspond to RMV’s MRI knee method and has been applied to 119 males with ages in the 8 intervals $14-14.5, 15-15.5, \dots , 21-21.5$ (curiously excluding all males with less than 6 months to their next birthday). Using the data from the table on page 59 of the report and applying the same computational procedure as for Lucas et al. [9], we obtain parameters listed in Table 2. Socialstyrelsen [18] cannot be seen as a validation of the RMV procedure, as only the knees of each person is examined and different assessors are used. However, one might expect the results to be fairly relevant for the RMV procedure.

The largest study so far on an MRI knee indicator similar to that used by RMV is Ottow et al. [12]. Five different stages of the age indicator occur in the study: IIc, IIIa, IIIb, IIIc, and IV. Table 3 in Ottow et al. [12] lists the number of males in the study population for which each indicator value has been observed, and also gives summary statistics for the ages within each group. We use from these the minimum, maximum, and the three quartile values. With these values as starting point, we reconstruct raw data in a similar way as for the DARLInG data and apply maximum likelihood estimation.

As mentioned in the introduction, a small subset of RMV’s cases has undergone second opinion. In these, more than half of the knees assessed as mature by RMV was deemed immature in the second assessment. One cannot draw general conclusions from such a small and selected subpopulation, but a worst case scenario might be that around half of all knees that are “almost mature” (i.e., stage IIIc) are incorrectly classified as mature in RMV’s material. Based on that possibility, we have also estimated parameters under the assumption that half of those 32 observations classified in Ottow et al. [12] as stage IIIc are counted together with stage IV (“mature”).

Comparing the results in Table 2, we see that results based on Socialstyrelsen [18] and Ottow et al. [12] are quite similar, while the ajdusted results predictably show somewhat earlier maturation age. However, these studies are limited in size, and given the large variation observed in parameters for third molar maturation, it seems dangerous to draw to firm conclusions about knee maturation parameters based on only two studies. Thus, we will use the prior

24 $$\begin{array}{@{}rcl@{}} \pi_{K_{\text{MAIN}}}=(\theta_{21},\theta_{22}) &\propto& \operatorname{Normal}(\theta_{21}; 18.5, 0.8) \cdot\\&&\operatorname{Normal}(\theta_{22}; 1.4, 0.4)\cdot I(\theta_{12}>0).\\ \end{array} $$

This prior uses the same amount of uncertainty as the prior in Eq. 23, but centered on the values 18.5 and 1.4. The prior is illustrated in Fig. 1. Calling it *K*_MAIN_, we have also looked at results using priors *K*_HIGH_ and *K*_LOW_ which differ from *K*_MAIN_ in that the the expected value of *𝜃*_21_, 18.5, is replaced by 19.5 or 17.5, respectively.

### Specification of prior for the population profile

Specification of a prior age distribution for the population of males that have been subjected to RMVs age determination procedure during 2017 is a difficult task. The prior for an individual should be based on all knowledge about this individual excluding the age indicators. Such general knowledge can include other observations of biological maturity made by medical personnel, observations of psychological maturity made by teachers or other qualified observers, documentable circumstances surrounding the life situation, as well as of course the reasons why the person has been required by the Swedish migration authority to complete the RMV procedure. The age prior for the whole population studied in this paper should represent an average over their individual priors.

The difficulties with establishing such a prior can lead some researchers to the conclusion that frequentist statistical methods where a prior does not seem to be needed are preferable to our Bayesian approach. However, when conclusions are drawn using such frequentist methods, they generally correspond to the use of a particular prior, as mentioned in the introduction. For example, the “hidden” prior assumption may be that the a priori age distribution corresponds to that of a study population, or that it is uniform within some age interval, for example between 14 and 25. So, the relevant question is whether we can establish a prior that is more realistic than such hidden priors.

**Fig. 2:**
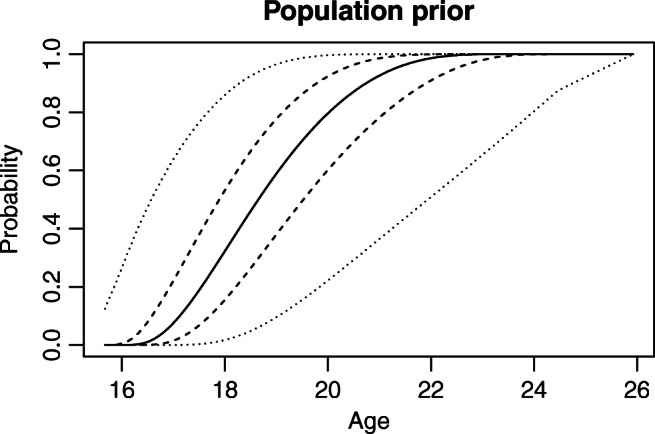
The prior age profile model. The continuous line represents the most likely age profile. It shows, at each age, the proportion of the population below that age. The dashed lines represent 50% credibility intervals at each age for the proportion in the population having this age or less. The dotted lines represent a 95% credibility interval

In this paper, we will use as a starting point an age profile illustrated in Fig. 2. This line represents the cumulative prior probability that a person has an age below that given on the x-axis. Thus, for example, the prior probability that a person is below 18 years is about 35%. Mathematically, the prior is represented by a gamma density with parameters 4 and 1, translated to be at least 15 and truncated to the interval between 15 and 30. As this distribution is rather arbitrarily chosen, we use a hierarchical prior with a lot of uncertainty around this starting point. First, we discretize the age variable into *T* = 100 possible ages (see Stochastic model” for details). Then, we model the proportions of the population at each age with a Dirichlet distribution depending on a hyperparameter *α* (see Eq. 8). Essentially, if *α* is close to zero, any combination of proportions is almost equally likely, and the prior imposes no structure on the possible age profile. If *α* is large (say, 1000) only age profiles that have almost equal proportions at each discrete age are possible. This forces any age profile to look almost as the starting point profile mentioned above. We use the compromise value *α* = 3. As can be seen in Fig. 2, 95% credibility intervals for the prior percentage of persons that have reached specific ages are then quite wide. Thus, we believe the prior is flexible enough so that our choice of starting point will not influence results significantly; this is further discussed in the supplementary material [11].

### Convergence and accuracy

The MCMC simulation outlined in “Stochastic model” is a Metropolis-within-Gibbs algorithm, using Gibbs sampling for *ψ* and *τ* and proposal functions for *𝜃*_1_ and *𝜃*_2_, the tooth and knee model parameters, respectively. We use symmetric proposal functions, perturbing the four parameters *𝜃*_11_,*𝜃*_12_,*𝜃*_21_, and *𝜃*_22_ using normal distributions (see the R code for details). Our acceptance rates were around 0.3, while the autocorrelations unfortunately became quite high.

However, as the algorithm outlined above was straightforward to implement, we have chosen to stick with it rather than work on more complex MCMC proposal functions. Indeed, with clearly unimodal posteriors, convergence is fairly easy to assess using plots and multiple chains with different starting points. For final results, we simulated 1 million MCMC cycles for each model, using a burn-in of 20000 cycles. Each such computation took around 20 min on a laptop. The R code used is available from the supplementary material [11].

## Results

Using the main prior presented in “Age indicator model parameter values” and “Specification of prior for the population profile”, we obtained results shown in Table 3. Regarding the RMV procedure as a classification procedure for being above 18 years, we also obtain, in percent (with 95% credibility intervals in parentheses): sensitivity, 93 (86–98); specificity, 67 (39–94); positive predictive value, 93 (76–100); negative predictive value, 59 (7–92).

It may be more revealing to look at the consequences of the RMV procedure when individuals are sorted into the categories of Table 1. Table 4 shows the percentage of children in each of the categories. Note in particular, the percentage of children among those with mature teeth and immature knees, and vice versa. These are classified as adults with the RMV procedure.

**Table 4:** Percentage children in each category (95% credibility intervals in parentheses)

	Knees mature	Knees immature	No data knees	Sum
Teeth mature	**1 (0–8)**	**24 (8–78)**	**2 (0–9)**	3 (0–12)
Teeth immature	**19 (1–64)**	63 (8–95)	28 (2–70)	36 (4–74)
No data teeth	**5 (0–17)**	48 (4 –88)	7 (0–22)	11 (1–27)
Sum	6 (0–23)	53 (6–90)	9 (1–26)	15 (1–34)

The cells with bold values represent those where the RMV procedure classifies males as adults

**Fig. 3:**
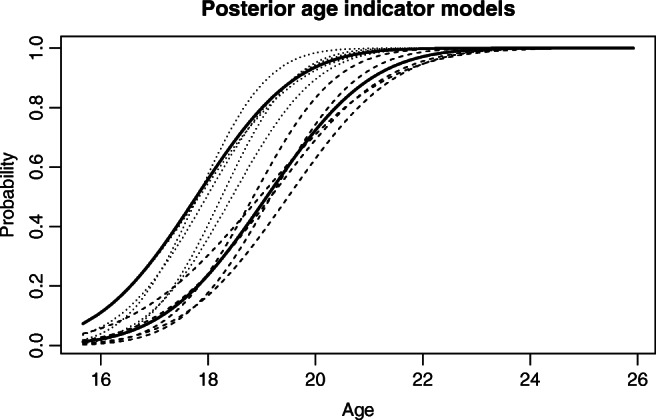
The posterior probability for observing mature teeth (right bold line) or knees (left bold line) as a function of age, assuming the observation is not missing. The two bold lines represent the most likely models. The 5 dashed lines represent other possible posterior models for teeth maturity, while the 5 dotted lines represent possible posterior models for knee maturity

**Fig. 4:**
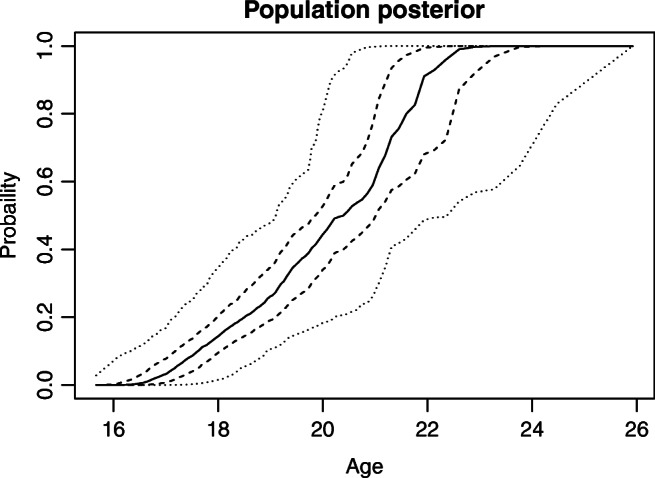
The posterior age profile model. The continuous line represents the most likely age profile. It shows, at each age, the proportion of the population below that age. The dashed lines represent 50% credibility intervals at each age for the proportion in the population having this age or less. The dotted lines represent a 95% credibility interval

The posterior expectations for the age indicator model parameters are not very different from the prior expectations: The prior and posterior expectations are 19.5 and 19.4 for *𝜃*_11_, 1.6 and 1.6 for *𝜃*_12_, 18.5 and 18.1 for *𝜃*_21_, and 1.4 and 1.5 for *𝜃*_22_, respectively. However, the uncertainty around these expectations is reduced (see Fig. 3). Note also that average posterior estimates for *𝜃*_13_ and *𝜃*_23_, the proportion of persons with no observable maturity for their tooth or knee at 20 years, respectively, are 0.18 and 0.04. The proportion seems to increase slightly with age for teeth (with *𝜃*_14_ averaging 0.02) but be fairly stable for knees (with *𝜃*_24_ close to zero). In the posterior model, *𝜃*_11_ − *𝜃*_21_ averages 1.36, with 1.18,1.53 as a 50% credibility interval and 0.85,1.9 as a 95% credibility interval. Thus, it is clear that knees on the average mature earlier than teeth (see the next section for a further discussion of this difference). Finally, Fig. 4 illustrates the posterior population age distribution. Comparing it with Fig. 2 ,we see the posterior population profile is substantially shifted towards higher ages. The uncertainty is also smaller.

### Robustness

In any Bayesian analysis, it is important to study how reasonable changes to the prior may affect conclusions. In the present context, this is particularly important, as the only firm data we use are those of Table 1; all other information is input using the prior. For example, the fit of the data will not be influenced if we change the age indicator models by simply translating the ages they refer to and at the same time correspondingly translate the ages of the population age prior.

In the supplementary material [11], we have studied the effects of changes in both the age indicator prior and the population age profile prior. Here, we focus on studying the effect on some of the main conclusions from the previous section. Table 5 shows results for the two most interesting classification error rates from Table 4. An interpretation of these results is that the rates are probably around 20–25%, but they may also be either higher or lower.

**Table 5:** The dependenceof two classification error rates on choices of priors: We look at the percentage of children among those classified with mature knee and immature teeth (left) and among those classified with immature knee and mature teeth (right)

	Percent children within classification K+, T−			Percent children within classification K−, T+		
	*K*_LOW_	*K*_MAIN_	*K*_HIGH_	*K*_LOW_	*K*_MAIN_	*K*_HIGH_
*T*_LOW_	41 (5–87)	30 (2–81)	19 (1–64)	46 (3–93)	37 (1–89)	29 (1–81)
*T*_MAIN_	35 (3–82)	19 (1–64)	13 (0–48)	40 (2–91)	24 (0–78)	18 (0–67)
*T*_HIGH_	23 (1–72)	13 (0–51)	7 (0–33)	26 (0–84)	16 (0–68)	11 (0–53)

Results for our main priors are in the centers of the tables; other results are for various combinations of the priors *T*_LOW_,*T*_MAIN_, and *T*_HIGH_ for teeth and *K*_LOW_,*K*_MAIN_, and *K*_HIGH_ for knees. The parentheses contain 95% credibility intervals

**Fig. 5:**
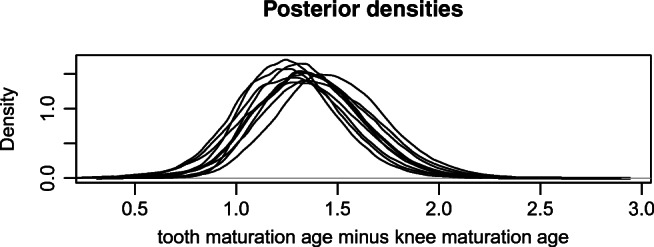
The posterior density for *𝜃*_11_ − *𝜃*_21_, i.e., the difference between the age at which 50% of males have mature teeth and the age at which 50% of males have mature knees. The bold line is the density under our main prior. The other lines represent posteriors under other combinations of priors (the same combinations as those used in Table 5)

Finally, Fig. 5 shows the distribution of the difference *𝜃*_11_ − *𝜃*_21_ under different priors. The figure establishes very clearly that, even if we vary the priors as specified, the diffe- rence is roughly between 1 and 1.5 years. Note that this is the case even when we use the priors *T*_LOW_ and *K*_HIGH_, where the difference apriori is negative. Thus, it is clearly estab- lished that the data of Table 1 is incompatible with models where the knee on average matures later than the teeth.

## Discussion

In this paper, we present a general method for studying the properties of an unvalidated age assessment procedure, and the consequences of its use. Although uncertainties in many results are large, we show how it is possible to obtain some information about model parameters and error rates using only classification counts and priors guided by published studies. A key reason that results are possible to obtain is that two age indicators have been observed, so that one can learn about the differences in their parameters. Applying our model in a situation with three or more age indicators would probably further strengthen results.

Parts of our results are formulated viewing the RMV procedure as a classification method for classifying people as above or below 18 years. This should in no way be interpreted as support for the idea that medical age assessment should be viewed as a classification procedure. Instead, we believe that medical age assessment should produce information about age specifying both the range of likely ages and the uncertainty in the information. Such information can then be combined with uncertain information from other sources. Indeed, within most legal systems, there is a requirement to weigh all relevant information together before making a legal decision about age. We hope to return with a different paper specifying how we propose computations in connection with medical age assessments can best be done. Indeed, in spite of the issues we raise with the RMV procedure, we believe medical age assessment can be a well-functioning tool when observation methods are properly selected and validated and computational methods are selected and performed correctly. But in this paper, the focus has been to study the consequences of the procedure, which in practice has functioned as a classification rule in Sweden.

According to our findings, about 33% of all male children that have been subjected to the RMV procedure have been erroneously classified as adults (i.e., the specificity is about 0.67). Conversely, the sensitivity of about 0.93 means that about 7% of male adults have been classified as children. Some other findings are summarized in Table 4, which shows that error rates are quite substantial for several of the 9 categories the RMV procedure classifies into. Although the chance of classifying adults as children is very high in some cases (for example about 72% for those with immature teeth and no data for their knee), we would argue that it is the risk of classifying children as adults that is the most serious issue, as such a misclassification has the most serious consequences for the individual. Our findings show that about 19% of those classified with a mature knee and immature teeth are children, as are about 24% of those classified with an immature knee and mature teeth. Taking into account the uncertainty that surrounds our choice of priors, Table 5 supports the claim that we can be confident these rates are in the interval 10–40%.

An important finding is that the age at which 50% of all males have a mature knee appear on average about 1–1.5 years before the corresponding age for teeth, when measured with the RMV procedure. The robustness of this claim is illustrated in Fig. 5, which shows that the conclusion is fairly insensitive to reasonable changes in the priors.

In addition to the influence from choices of parameters in our priors, our results are of course also influenced by our choice of model. We have already mentioned that we assume conditional independence of the age indicators given age. We also assume a particular shape of the maturity function in Eq. 2; further studies may refine such choices. Finally, we have made no attempt to adjust for the fact that the asylum seekers the RMV procedure has been applied to generally have different genetic and socioeconomic backgrounds compared to the study populations of most age indicator studies.

Generally, there is a large uncertainty in our numerical results. This is an unavoidable consequence of the fact that the only firm data available on the RMV procedure are those of Table 1. As this procedure has determined the fate of thousands of young people, and continues to do so, there is an urgent need for a proper validation to provide more data.

## Footnotes

1
https://www.rmv.se/verksamheter/medicinska-aldersbedomningar/metoder/

2 The exact wording of the conclusions produced by RMV have several forms. However, as these conclusions are then mapped by the Swedish migratory authority to decisions about age, the exact wording is of little consequence. We will in this paper simply refer to assessment of above or below 18 years.

3
https://www.svd.se/rmv-andrar-aldersbedomning-efter-granskning

4 The tests are not mandatory and only those who have not been able to make their under-age plausible are offered the tests. However, if they then do not agree to take the tests, they will most likely be assessed as adults.

5 In fact, the table lists those that are classified as above/below 18 years according to the method ST-DA [9]. However, as the classification is (mostly) based only on the left mandibular third molar and the database UK-caucasian [22], it should correspond directly to classification in terms of stage H of Demirjian’s scale.

6 Contrary to the other data we discuss, this data pools both males and females.
